# Complete Genome of a Genotype I Japanese Encephalitis Virus Isolated from a Patient with Encephalitis in Vientiane, Lao PDR

**DOI:** 10.1128/genomeA.00157-12

**Published:** 2013-02-21

**Authors:** Fabien Aubry, Manivanh Vongsouvath, Antoine Nougairède, Rattanaphone Phetsouvanh, Bountoy Sibounheuang, Rémi Charrel, Sayaphet Rattanavong, Koukeo Phommasone, Onanong Sengvilaipraserth, Xavier de Lamballerie, Paul N. Newton, Audrey Dubot-Pérès

**Affiliations:** a Aix Marseille Université, Institut de Recherche Pour le Développement (IRD), École des Hautes Études en Santé Publique (EHESP), UMR_D 190 Emergence des Pathologies Virales, Marseille, France; b Lao-Oxford-Mahosot Hospital, Wellcome Trust Research Unit (LOMWRU), Microbiology Laboratory, Mahosot Hospital, Vientiane, Lao PDR; c Centre for Clinical Vaccinology and Tropical Medicine, Nuffield Department of Clinical Medicine, University of Oxford, Churchill Hospital, Oxford, United Kingdom

## Abstract

Japanese encephalitis virus (JEV) (*Flaviviridae*, *Flavivirus*) is an arthropod-borne flavivirus transmitted by *Culex* species mosquitoes. We report here the complete genome of the JEV genotype I strain JEV_CNS769_Laos_2009 isolated from an infected patient in Vientiane, Lao People’s Democratic Republic (PDR) (Laos).

## GENOME ANNOUNCEMENT

Japanese encephalitis virus (JEV) (*Flaviviridae*, *Flavivirus*) is an arbovirus transmitted through a zoonotic cycle involving *Culex* species mosquitoes, pigs, and water birds (1, 2). JEV infection in humans is an epidemiological cul-de-sac, associated with significant mortality and frequent neurological sequelae. Despite possible prevention through vaccination, JE remains a serious public health concern in Asia (2, 3).

JEVs are divided into five genotypes based on the nucleotide sequences of the structural genes (4–7). The first JE case was reported in Japan in 1871, but the virus was not sequenced until 1987 (8). One hundred thirty-nine full-length genomic sequences are available in GenBank, including those of genotypes I to IV (strains isolated from different vectors or hosts in Asia and Australia [9–16]) and two genomes of the genetically distant genotype V (7, 17, 18); 33 genomes originate from human isolates, including only 3 in genotype I (10).

In June 2009, strain JEV_CNS769_Laos_2009 was isolated in Vero cells from the cerebrospinal fluid (CSF) of a patient admitted with encephalitis at Mahosot Hospital, Vientiane, Laos. This 12-year-old boy died after being admitted from Meuan District, Vientiane Province (18.405 N, 101.956 E; 243 m above sea level [msl]), with 3 days of fever, headache, vomiting, diarrhea, confusion, convulsions, neck stiffness, and a Glasgow Coma score of 11 of 15. The CSF white count was 335/ml (76% neutrophils, 24% lymphocytes). Viral RNA was extracted from cell culture supernatant at passage 3. Overlapping real-time PCR (RT-PCR) products spanning the complete coding sequence were obtained using primers designed from (i) the alignment of 8 complete open reading frames (ORF) of genotypes I, III, and V (GenBank accession no. EF571853, AY508812, GQ902063, GU205163, HM366552, HM228921, GQ902062, and JF915894) (6, 7, 18) and (ii) the sequences established from the resulting amplicons. The complete noncoding regions were amplified and sequenced following the uncapping and circularization of the viral RNA using a tobacco acid pyrophosphatase and an RNA ligase.

The complete genome of JEV_CNS769_Laos_2009 is 10,965 nucleotides (nt) in length. The ORF (10,299 nt) encodes a polyprotein processed into three structural proteins, capsid (C) (127 amino acids [aa]), premembrane/membrane (prM/M) (167 aa), and envelope (E) (500 aa), and seven nonstructural proteins, NS1 (352 aa), NS2A (227 aa), NS2B (131 aa), NS3 (619 aa), NS4A (149 aa), NS4B (255 aa), and NS5 (905 aa). The 5′ and 3′ noncoding regions (NCRs) are 96 and 570 nt long, respectively. Cleavage sites are identical to those reported previously, and 4 glycosylation sites (amino acid positions 142, 448, 924, and 1001 in the prM, E, E, and NS1 proteins, respectively) are predicted (19).

This constitutes the first isolation and complete characterization of a clinical strain of JEV in Laos. Phylogenetic analysis revealed that JEV_CNS769_Laos_2009 belongs to genotype I, which is consistent with previous data from neighboring countries (5, 7). The nucleotide and amino acid sequences of the ORF of the JEV Laos strain are 99.1% (10,202/10,299 nt) and 99.9% (3,430/3,433 aa) identical to those of strain GSBY0861 (GenBank accession no. JN381833), isolated from a mosquito in China in 2008 (6). The noncoding regions of both strains are almost identical, with a difference of only 2 nucleotides in the 3′ NCR.

This full-length sequence might contribute to a better understanding of the molecular epidemiology of JEV and to the study of various aspects of JEV biology.

### Nucleotide sequence accession number.

The GenBank accession no. of JEV_CNS769_Laos_2009 is KC196115.
